# A thermally robust method of sample sealing for capillary DLS

**DOI:** 10.1016/j.mex.2023.102142

**Published:** 2023-03-20

**Authors:** Sharla Harvey, Jake Austin, Darrell Bancarz, Alex Malm

**Affiliations:** Malvern Panalytical Ltd., United Kingdom

**Keywords:** Capillary, Dynamic light scattering, Thermal ramps, *An optimized method of sample sealing for capillary Dynamic Light Scattering*

## Abstract

Capillary Dynamic Light Scattering (DLS), has recently been introduced as a simple and enabling technique that increases the measurement range of traditional DLS analysis with minimized sample volumes (Ruseva et al., 2018). The previously published protocol for the preparation of samples for analysis within a capillary called for sealing of the capillary end using a clay compound (Ruseva et al., 2019). This material is not, however, compatible with organic solvents, nor with elevated sample temperatures. To extend the uses of capillary DLS to more complex assays like thermal aggregation studies, a new sealing method is demonstrated using a UV curing compound. This further motivates the use of capillary DLS to minimize volumes of destroyed precious samples in pharmaceutical development assays to study thermal kinetics.

• Use of UV curing compound to seal capillaries used in DLS to preserve low volumes of sample.

## Graphical abstract

**Figure:**
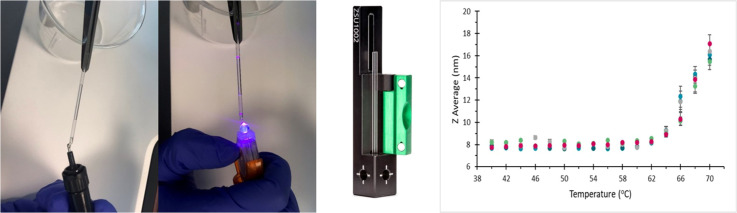

Specifications table

**Table:** 

Subject Area:	Materials Science
More specific subject area:	*Dynamic light scattering*
Method name:	*An optimized method of sample sealing for capillary Dynamic Light Scattering*
Name and reference of original method:	*‘Capillary dynamic light scattering: continuous hydrodynamic particle size from the nano to the micro-scale’, V Ruseva, M Lyons, J Powell, J Austin, A Malm, J Corbett, Submitted COLSUA (2018)*[1]
Resource availability:	*Capillary cell, UV bonding compound/resin (with UV curing light), Zetasizer Lab or Zetasizer Ultra*

## Method details

Dynamic light scattering (DLS) characterizes the temporal autocorrelation of coherent light scattered by dispersions of diffusing particles or molecules. The relaxation times inferred from this autocorrelation can then be used to deduce ensemble mean diffusion coefficients which can then be converted to hydrodynamic particle size using the Stokes Einstein equation. This particle size information can be used independently or in conjunction with intensity measurements from static light scattering (SLS) measurements to monitor structural or conformational changes of dispersed material, with sensitivity to aggregation due to the 6th power relationship between light scattering intensity and particle radius. Such stability assays may be accelerated via the application of a stress to the sample through either mixing, freeze-thaw or through measurements at elevated temperatures [3]. While such a measurement can be informative, the destructive nature of a temperature trend measurement drives the requirement for analytical methods to use as small a sample volume as possible. Sample presentation in a batch light scattering apparatus is typically via cuvettes, which are available in a range of form factors for different applications. Whilst low volume plastic cuvettes combine low volume and contamination free disposability, e.g., ZEN0040 (Malvern Panalytical), their integrity to high temperatures is a shortcoming. Glass or quartz cuvettes offer superior optical quality and temperature robustness. However, these can be difficult to clean following the thermal aggregation of a sample, and while cuvettes such as the ZEN2112 can use a sample volume as low as 12 µL, the internal volume of such a cuvette means that a much larger volume is required to compensate for sample evaporation during a temperature trend.

While options exist to use low volumes of sample in a sealed well plate format, the temperature variability in such a setup can be significant and optical quality generally not as good as for a cuvette-based measurement [4]. Samples may be sealed within a cuvette or well based format using oil, however this can lead to complications due to interactions between the oil-water interface, especially in complex formulations comprising hydrophobic groups.

Prior to this work, sample presentation via a capillary introduced a low volume, high quality measurement option for DLS and light scattering measurements. However, the capillary sealing methods explored at the time meant that a limited temperature range was accessible using this approach.

The method presented here demonstrates an approach to collecting temperature trend light scattering data without having to compromise by using higher sample volumes or a constrained temperature range.

The capillary sealing method presented is also demonstrated for other volatile sample measurements, such as materials dispersed in organic solvents.

## Method development

### Clay with PTFE

The capillary sealing method in [2], describes the use of a clay which is conveniently used by pushing the end of the capillary into the clay such that a “slug” of clay fills the open end of the capillary. This clay melts at around 40 °C, meaning that during a temperature trend, the sealing of the capillary is not effective with the melted clay either contaminating the sample or allowing the sample to escape from the capillary.

**Fig. 1:**
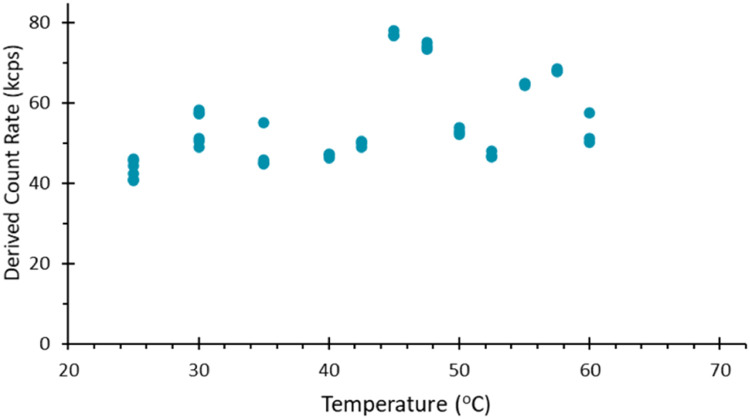
Derived mean count rate as a function of sample temperature for a 1 mg/ml dispersion of BSA, recorded for a capillary sealed with clay. The onset of fluctuations in the detected scattering demonstrates the breakdown of the clay compound, most notably after 40 °C. During this experiment, the intended end temperature was 70 °C. However, above 60 °C, the sample was lost, and no further data was recorded.

To create an inert barrier between the clay and the sample, a trial was conducted with a piece of PTFE tape laid across the sealing clay when the capillary was pushing into the clay. The capillary punches out a piece of PTFE tape which is then sealed in place by the clay beneath it. Using this method during a temperature trend with a BSA sample, data was recorded beyond 40 °C. However, the data recorded above 40 °C was erratic despite being below the aggregation temperature of the sample. The sample was eventually lost with no usable signal detected beyond 60 °C, indicating that the seal was compromised, and fluid was no longer present in the detection volume of the instrument, Fig. 1.

### Wax

**Fig. 2:**
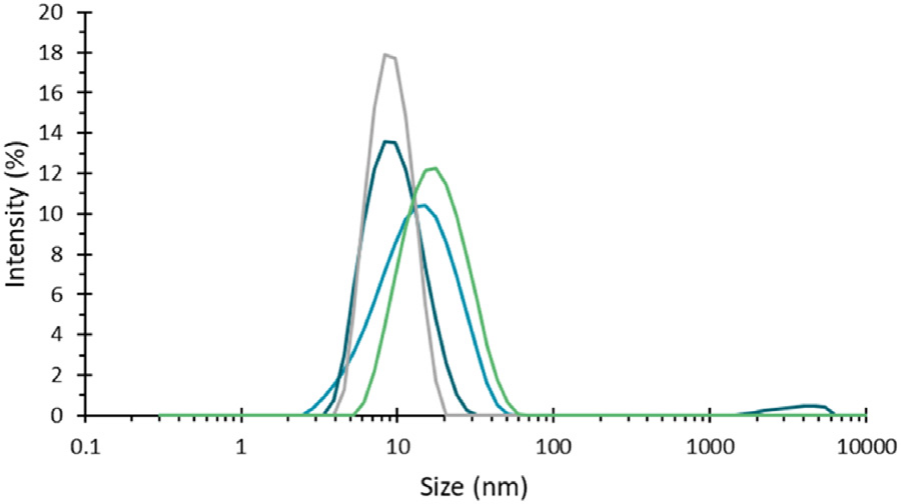
Intensity weighted particle size distribution for a 1 mg/ml dispersion of BSA, recorded in several capillaries sealed using wax. All these measurements were performed at 25 °C such that no sample aggregation was expected. However, shifts in peak size and presence of larger material is evident, suggesting that the heat used to melt the wax during sealing has influenced the sample.

A wax sealing method was tested whereby a heated tool is used to melt a small pool of wax, in which the capillary is dipped. The heated tool can also be used to manipulate a small piece of wax to create the seal of a capillary. The potential interaction between samples and the wax, and/or the effect of the heated tool being used close to a fragile sample such as a protein did show some effects on samples during trials of this method (Fig. 2). While the wax has a higher melting temperature than the sealing clay, 99 °C, sealing efficiency was compromised in trials at temperatures typical of a thermal stress study. For both reasons, the wax sealing method was rejected.

### 3D printing pen

Filament deposition manufacturing (FDM) 3D printers typically use a PLA or ABS filament material, with an extrusion temperature of 180–200 °C. Handheld filament extruders or “pens” are commercially available for extruding controlled quantities of polymer for model making applications. The extrusion temperatures of these filaments mean that these could be used for creating a seal whilst also being robust to the temperatures experienced during a light scattering measurement.

Some successful trials of this technique were achieved. However, it was noted that the process was very difficult, with several sealing attempts needed in some cases. The proximity of the hot extruder to a sample was also a cause for concern as per the wax sealing trials. Whilst this technique could be used, it was therefore abandoned within this work.

### UV resin pen

Resins that cure under exposure to UV light are commercially available for a range of applications. Within the context of this work, a UV curing resin with a pen-like applicator was used. The application of this resin as a sealing compound was found to be straight forward in comparison to other methods. Prior to exposure to UV light, the resin is suitably viscous such that the deposited droplet can be controlled and positioned over the end of the capillary, whereas the wax or 3D printing filament, for example, could cool and harden before a sufficient seal is made.

**Fig. 3:**
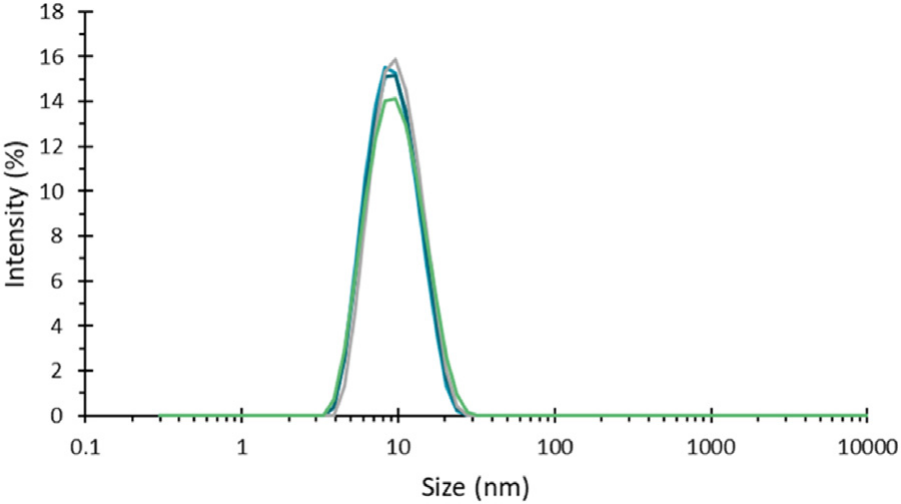
Intensity weighted particle size distribution for a 1 mg/ml dispersion of BSA, recorded in several capillaries sealed using UV curing resin, showing good quality repeatable data, with no apparent effect on the sample from either the resin or the UV light during sealing.

Good quality measurements of low scattering protein samples were gathered using this sealing method (Fig. 3), motivating a wider range of testing.

In isothermal measurements, where sample capillaries were sealed using clay, it was apparent that sealing only one end of the capillary was sufficient to stop movement of the sample slug within the capillary which was otherwise held in place by surface tension alone.

**Fig. 4:**
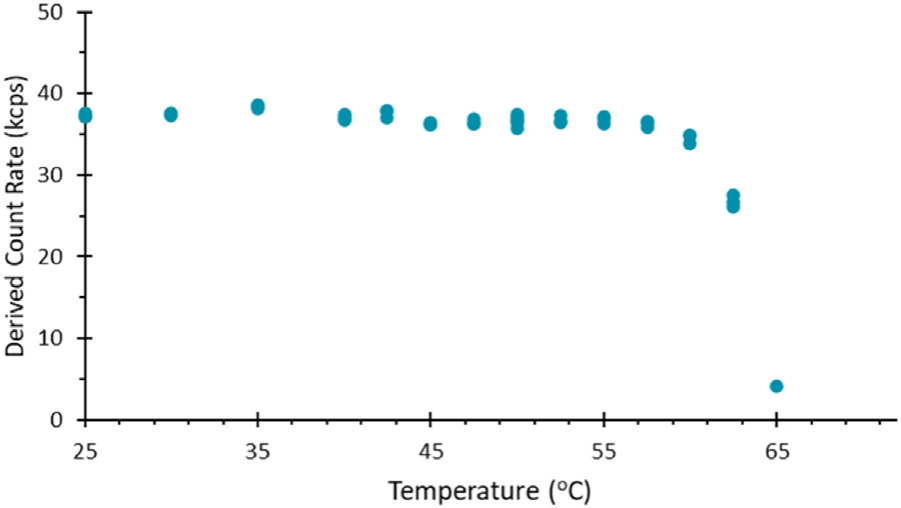
Derived mean count rate as a function of temperature for a BSA sample during a temperature ramp applied to a capillary where one end is sealed using UV curing compound. At elevated temperatures the scattering intensity.

In thermal ramp measurements of a sample where the capillary was only sealed at one end, it was however noted that there was a loss of measurement signal at elevated temperature. This is most easily demonstrated via the loss of scattering intensity (Derived Count Rate) as a function of temperature, Fig. 4. This therefore suggests that with increasing temperature, the slug of sample in a capillary may move if one end is sealed and the other is open to the atmosphere.

**Fig. 5:**
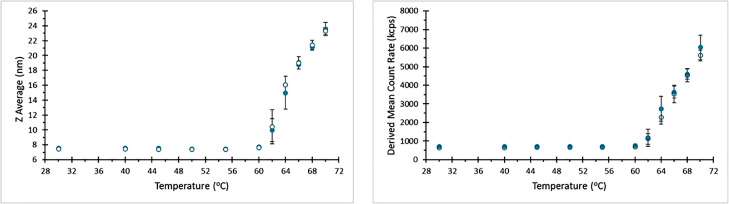
Comparison of thermograms for BSA recorded using a capillary sealed at both ends using UV curing resin (Filled symbols), and a low volume quartz cell (Hollow symbols).

When these measurements were repeated with capillaries sealed at both ends using UV curing resin, good quality thermograms were recorded for both hydrodynamic size and scattering intensity, showing good agreement with measurements in a conventional quartz cell, Fig. 5.

## Method protocol

### Sample preparation

A 3 mg/ml sample of bovine serum albumin (BSA) (Sigma Aldrich) in PBS buffer was prepared using a phosphate buffered saline tablet (Sigma) with 200 mL of ultrapure deionized water and filtered with 100 nm and 20 nm filters. For each thermal ramp a new aliquot was prepared from the same stock solution.

For chemical compatibility measurements, 200 kDa polystyrene standard was dispersed in toluene (Sigma).

To verify the thermal stability of the UV resin, a 38 nm silica sample (TM-50 Ludox) was dispersed in ultrapure water.

All samples were loaded into new capillaries which were subsequently sealed using the method described below. Capillaries were loaded into the ZSU1002 capillary holder as described in [2], ensuring that the sample was appropriately positioned. For comparative measurements, samples were also loaded into clean ZEN2112 low volume quartz cuvettes. Between measurements, these cuvettes were cleaned using Hellmanex, followed by a rinse with ultrapure water before being dried using compressed air.

### Repeatability testing

Five thermal ramps were run from 40 °C to 70 °C in 2 °C increments with a 60 s equilibration time on a Zetasizer Ultra blue label (4 mW He-Ne laser at 632.8 nm). Five replicate measurements were carried out at each temperature using side scatter detection. To check for repeatability between instruments, a further four thermal ramps were carried out across four different Zetasizer Ultra systems, including both blue and red label (10 mW HeNe laser at 632.8 nm) models.

### Reproducibility testing

Three aliquots of sample were each prepared and sealed by 3 different users. On a blue Zetasizer Ultra, a thermal ramp was run for each aliquot using the same method as detailed in the repeatability testing.

For comparison, 50 µL of the same sample was transferred into a ZEN2112 low volume quartz cell, and the method was repeated with five backscatter size measurements at each temperature.

### UV pen sealing method

**Fig. 6:**
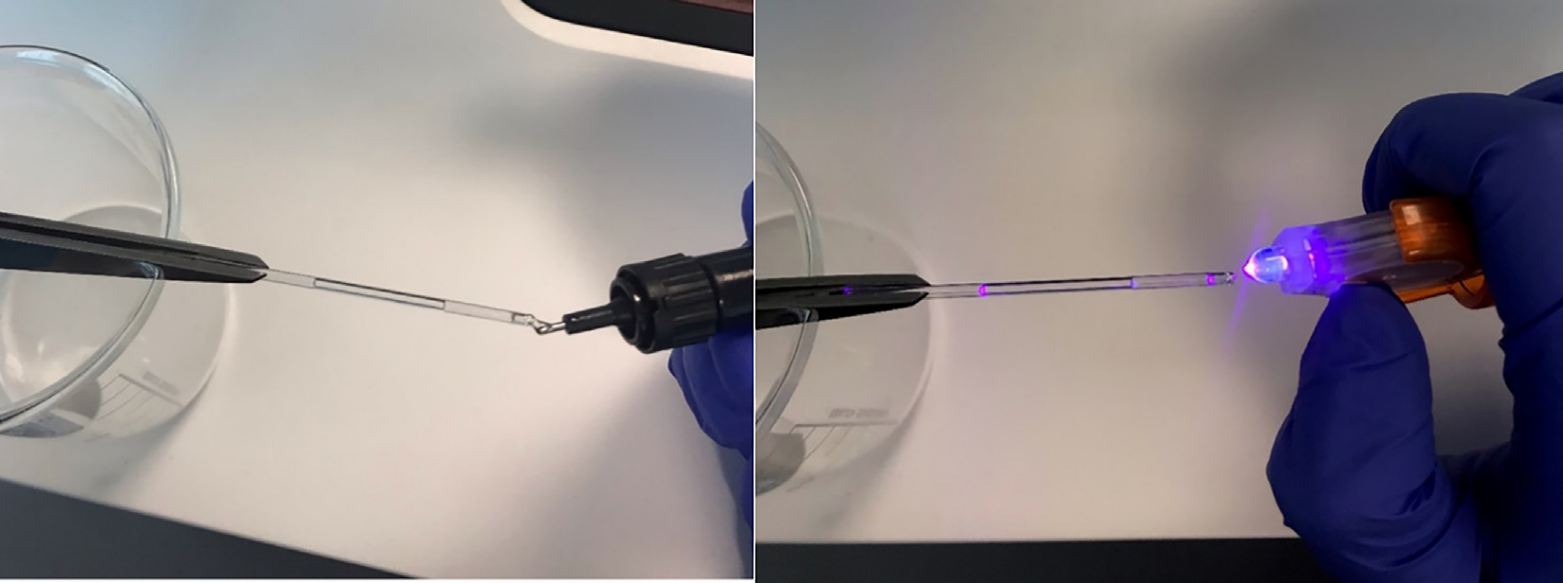
Left: Small amount of resin being administered onto the tip of the capillary. Right: UV light being used to set the resin which takes approximately 5 s.
• Switch on the UV light and hold the light close to, but not touching the tip of the capillary for around 5–10 s, until the resin has hardened, Fig. 6b.
• Repeat this process for the opposite end of the capillary to prevent sample evaporation.
• Wipe down the capillary to remove excess using a lint-free cloth and place into cell holder ensuring the sample is in the measurement window.

• Using tweezers to prevent any fingerprints, pick up a ZSU1002 capillary by one end.
• Dip the other end of the ZSU1002 capillary into your sample and allow it to wick up to approximately 1 cm up the capillary. This will equate to 10 µl of sample being loaded into the capillary. At least 3 µl of sample are required for reliable measurements.
• Hold the capillary horizontally and squeeze a small amount of resin onto the wet end of the cell until the tip is covered. It may travel up the cell by around 1 mm. Fig. 6a.

### Measurement SOPs

Particle size was recorded using the Adaptive Correlation process [5], with an automatic number of sub-runs captured for each measurement and automatic attenuator selection at each temperature point during the thermal ramp. Reported particle size distributions were calculated using the “General Purpose” mode of non-negative least squares analysis of the ZS Xplorer software.

## Method validation

**Fig. 7:**
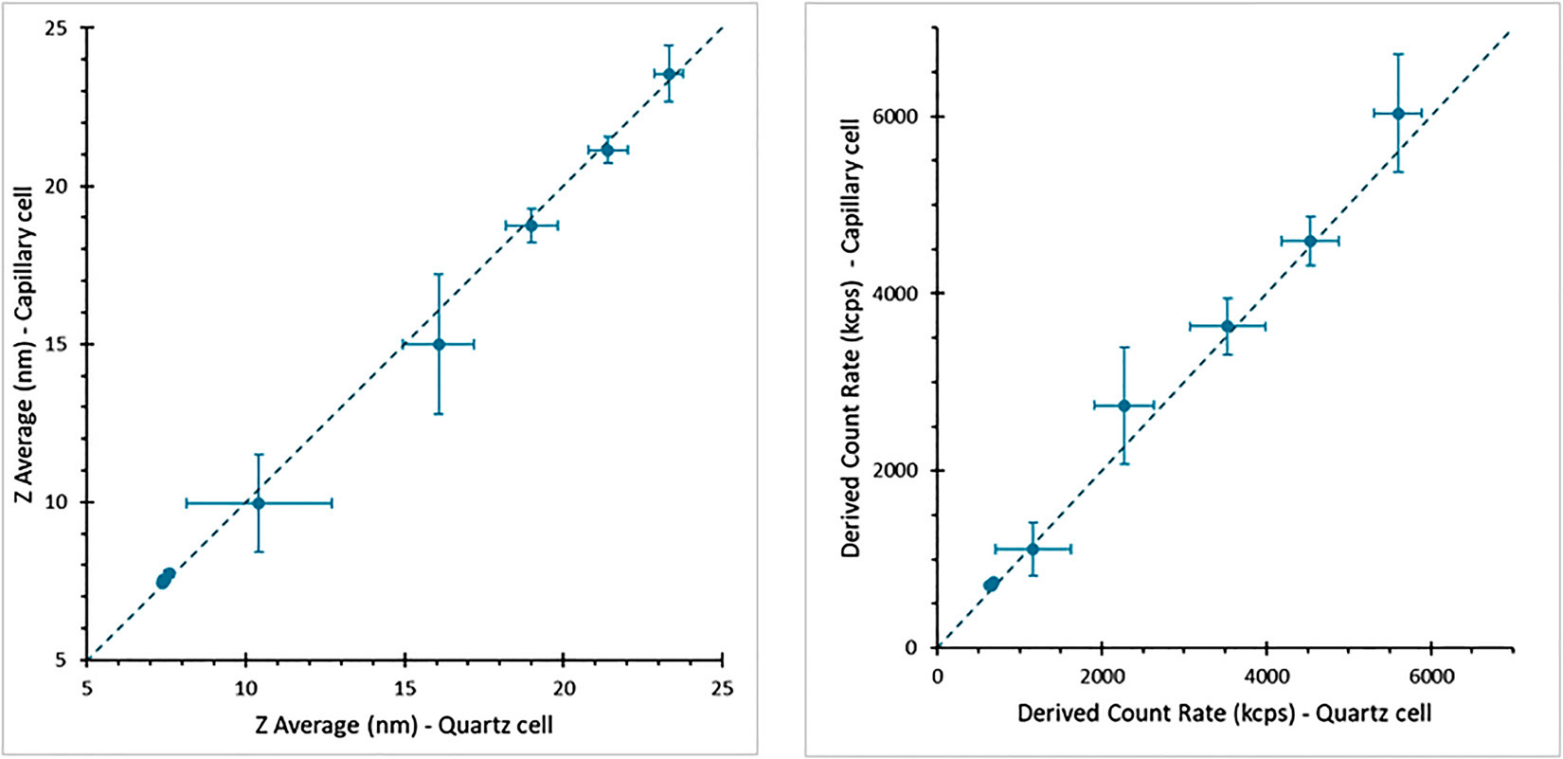
Comparison of reported size and derived count rate for thermal ramps of a BSA sample, recorded using a sealed capillary and a low volume quartz cell. Note that error bars increase significantly in scale during aggregation of the sample due to sample variability during this process.

In addition to the qualitative agreement between capillary sample thermograms and a low volume quartz cell, correlation of hydrodynamic size and scattering intensity was assessed by directly comparing the results from each method for repeat measurements, demonstrating good linear correlation throughout a thermal ramp, Fig. 7.

To further demonstrate the robustness and reproducibility of this method of loading and sealing capillaries using resin, comparisons were performed for multiple capillaries, multiple users/operators and multiple instruments.

## Multiple capillaries

**Fig. 8:**
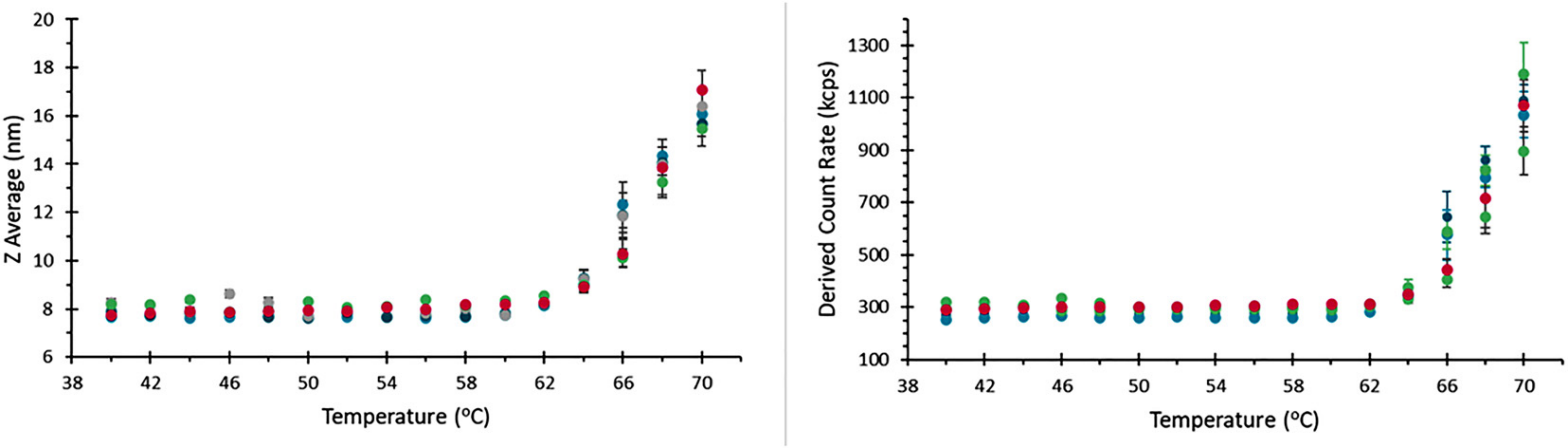
Thermograms for repeat measurements on a given instrument and user.

In repeats performed by a single operator with a single instrument, thermograms of the aliquots of BSA show good agreement with no apparent systematic difference between capillaries, Fig. 8.

## Users

**Fig. 9:**
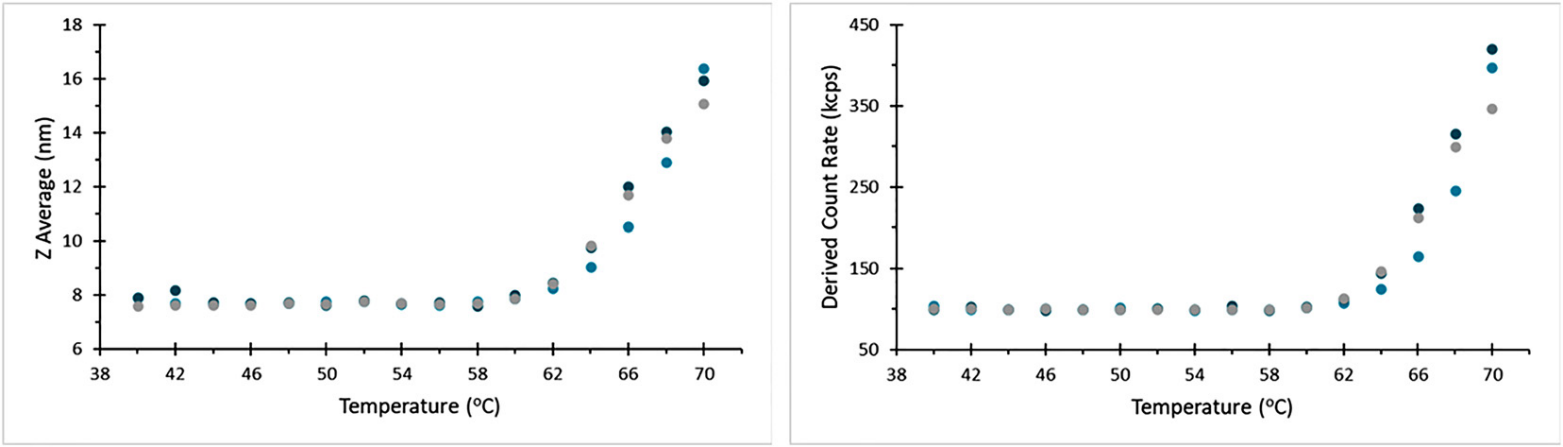
Thermograms recorded for samples loaded by separate operators, demonstrating good reproducibility.

Variation in thermograms recorded for samples loaded and sealed by different operators were also negligible, Fig. 9.

## Instruments

**Fig. 10:**
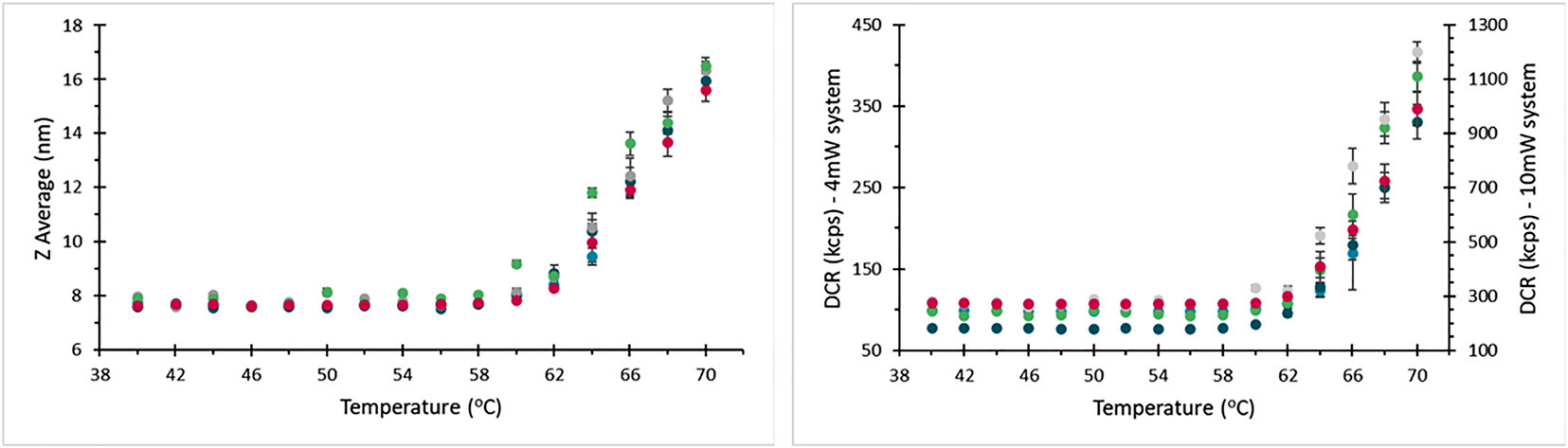
Thermograms recorded on different instruments, showing agreement between these measurements. Note the magenta data set here is recorded on a system fitted with a 10 mW laser, while the remainder of records here were for 4 mW systems, so the absolute light scattering between these systems is different, but the same trend is followed in each case.

Comparison of thermograms recorded using different instruments shows good agreement with respect to hydrodynamic size as a function of temperature. It is however notable that the derived count rate appears more variable than in other comparisons (Fig. 9). The absolute scattering intensity recorded using a given instrument of the same nominal specification may be affected by variations such as the absolute output power of the systems laser, efficiency of the detector, and transmission of optical fibers. This does not however affect the relative change in scattering intensity when a sample is stressed and undergoes aggregation, so while the baseline scattering may be different, the temperature at which this deviates and the rate at which it increases is comparable. The inclusion of a thermogram recorded with an instrument fitted with a higher power laser, plotted on the right-hand y-axis of Fig. 10b demonstrates this scaling well.

**Fig. 11:**
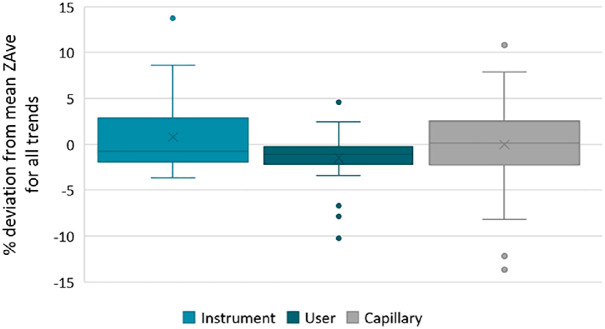
Deviation from an average thermogram for all temperature trend measurements for BSA, demonstrating variation based on instrument, user and capillary.

To demonstrate the degree of variability across all replicates of the above thermal trends, the Z Average particle size results for each temperature were averaged to calculate a mean overall trend for the aggregation of BSA. The residual to this average trend was then calculated as a percentage for all data points captured in the R&R study, and these are summarized in Fig. 11, grouped by variable.

This demonstrates the robustness of the sealing method as variation due to user is much narrower than variation across multiple capillaries or instruments. It should be noted that the widest variation across these measurements occurred after the aggregation point, and the baseline measurements at room temperature showed much less variation.

In the context of investigating aggregation behavior, all thermograms demonstrated an observable onset of aggregation at the same temperature increment of 60–62 °C.

During the review of this work, the stability of the resin used as a function of temperature was considered, and whether any thermal degradation of the resin may affect or contaminate a sample. This was explored via measurements of a silica sample at elevated temperature. The silica sample does not degrade because of temperature increase, so any measurable changes in the apparent size of the silica when dispersed and sealed in a capillary may be attributed to degradation of the resin.

**Fig. 12:**
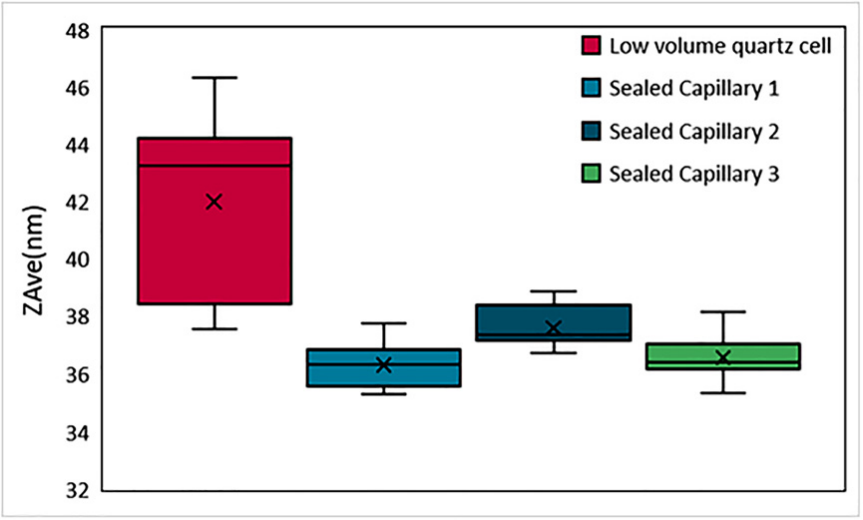
Measured average size of a silica sample, in a quartz cell and three separate sealed capillaries, with size measurements recorded at temperatures ranging from 25 to 90 °C.

Repeat measurements at a range of temperatures showed no trend or change in the apparent hydrodynamic size of the silica as a function of temperature in sealed capillaries. Comparative measurements in a low volume quartz cell showed more variability in this case, which was attributed to loss of sample due to evaporation, Fig. 12.

## Chemical compatibility

To explore the chemical compatibility of the UV curing compound as a capillary sealing method, capillaries were filled with a range of organic dispersants which were then sealed. Capillaries were examined after 1 min and 24 h to verify whether the sealed fluid was still in place (Table 1). All solvents trialed were held within the capillary for a short duration, however hexane and acetone were both shown to have leaked from their respective capillaries after 24 h. This therefore suggests that the capillary sealing method using UV resin may be used for commonly used solvents if measured directly after sample preparation, and most samples may be preserved in a capillary sealed using resin.

**Table 1:** Results of whether samples of different organic solvents showed compatibility with a UV curing resin after 1 min and 24 h.

Organic solvent	Compatibility after 1 min	Compatibility after 24 h
Acetone	Yes	No
Ethylene glycol	Yes	Yes
Heptane	Yes	Yes
Methanol	Yes	Yes
Ethanol	Yes	Yes
Methyl methacrylate	Yes	Yes
Isopropanol	Yes	Yes
Toluene	Yes	Yes
Hexane	Yes	No

**Fig. 13:**
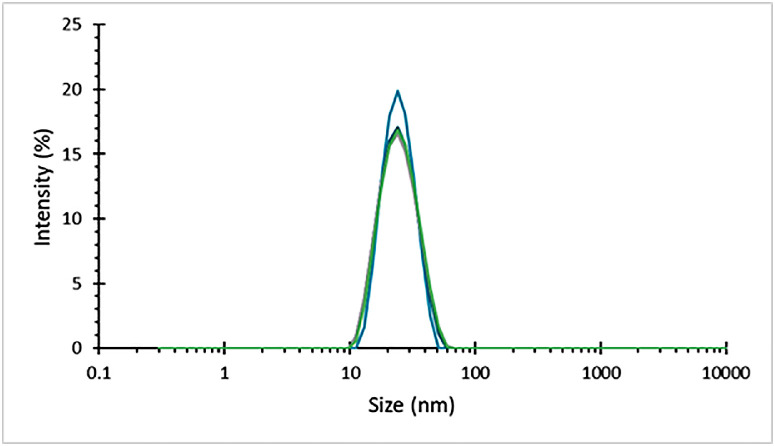
Intensity weighted particle size distribution for 200 kDa polystyrene dispersed in toluene, in a sealed capillary, measured approximately 24 h after preparation of the capillary.

To further demonstrate the robustness of sealing capillaries with resin for an organic solvent, a 200 kDa polystyrene standard dispersed in toluene was loaded and sealed in a capillary and the particle size distribution measured after approximately 24 h since sample preparation, Fig. 13.

## Safety considerations

To use this sample loading and sealing method safely, the safety guidance advised by the manufacturer of the UV curing resin should be followed. The LED belongs to risk group 1 (blue light) and should not be directed on to an eye or looked into directly. Contact between the resin and the skin should be avoided.

While some care is required in using this method, our trials showed that it is easily transferable between users and can be accommodated in standard lab practices without difficulty.

## Conclusions

The UV curing resin sealing method described is a robust and repeatable method of sealing capillaries which allow a range of extended functionalities of capillary loaded samples for light scattering measurements. The integrity of the sealing method to temperature allows convenient high-quality measurements of ultra-low volumes which would typically only be possible with samples sealed within an oil. The chemical compatibility of the UV resin also allows the use of the capillary cell for measurements other than those in aqueous buffers, meaning that a wider range of measurements can benefit from the benefits of capillary DLS.

## Declaration of Competing Interest

The authors declare the following financial interests/personal relationships which may be considered as potential competing interests.

The authors are employees of Malvern Panalytical Ltd. but declare no other competing interests.

## Data Availability

Data will be made available on request.
